# Supplementary material for: Associations between Smoking and Alcohol and Follicular Lymphoma Incidence and Survival: A Family-Based Case-Control Study in Australia
Source: Cancers (Basel). 2022 May 30;14(11):2710. doi: 10.3390/cancers14112710 (PMC9179256; doi:10.3390/cancers14112710)
Supplement: Supplementary file 1 [file cancers-14-02710-s001.zip › cancers-1700198-supplementary.pdf]

SUPPLEMENTARY FIGURES

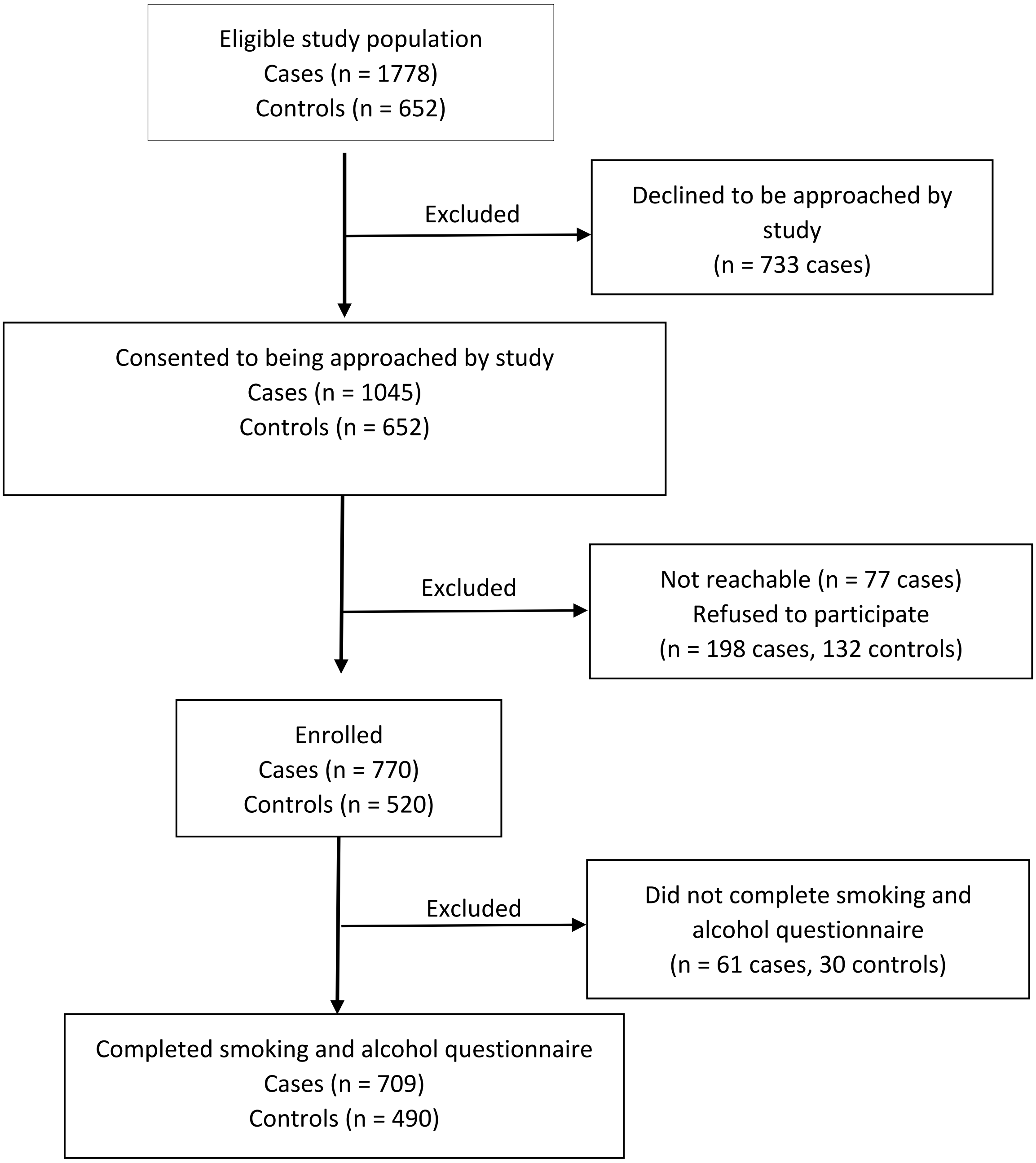

Supplementary Figure S1: Flowchart of recruitment to LEAF study

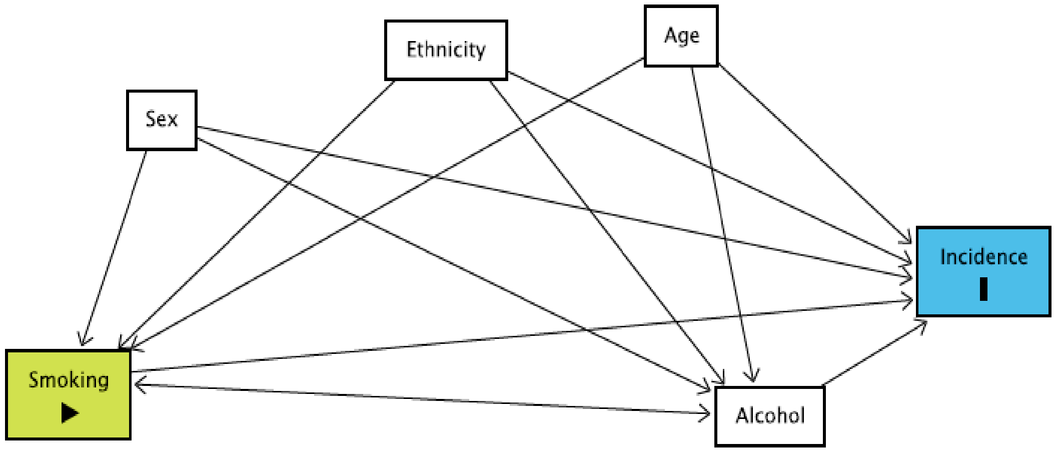

Supplementary Figure S2: Potential confounders of the association between smoking and FL incidence

Evidence underpinning associations included in directed acyclic graph (Supplementary Figure S2):

Smoking status and age – Smoking varies by age (1)

Smoking status and sex – Higher smoking prevalence in men compared to women (2)

Smoking status and ethnicity – Higher prevalence among whites compared to black population (3)

Age – Increased FL risk with age (4)

Sex – Increased FL risk in males compared to females (4)

Ethnicity – Increased FL risk in Caucasian compared to Asian or African population (5)

Alcohol intake – Inverse association with FL (6)

Alcohol intake and smoking – Smoking is significantly related to alcohol use (7, 8)

Alcohol intake and age – Alcohol intake varies by age (9)

Alcohol intake and sex – Alcohol intake higher in men than women (10)

Alcohol intake and ethnicity – Alcohol intake varies by ethnicity (11, 12)

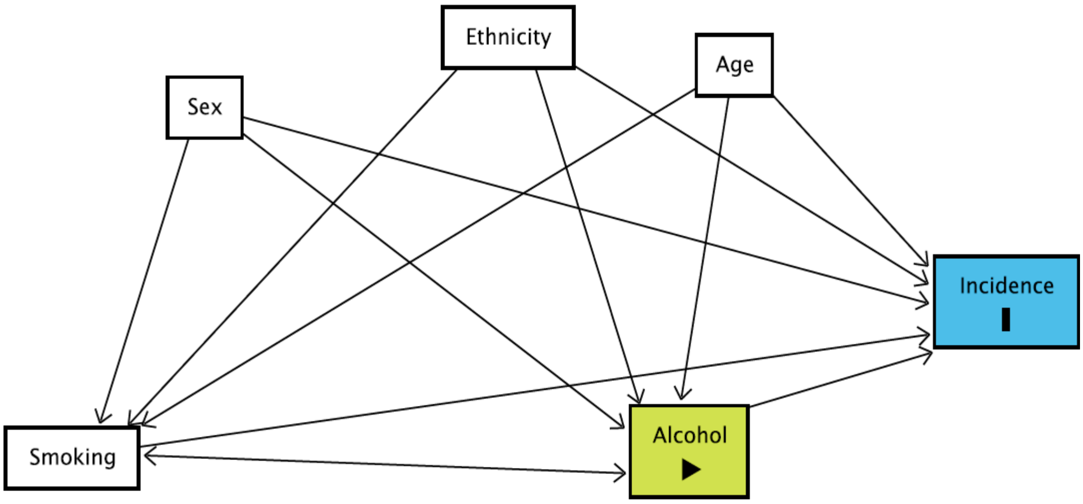

Supplementary Figure S3: Potential confounders of the association between alcohol and FL incidence

Evidence underpinning associations included in directed acyclic graph (Supplementary Figure 3):

As for Supplementary Figure 2.

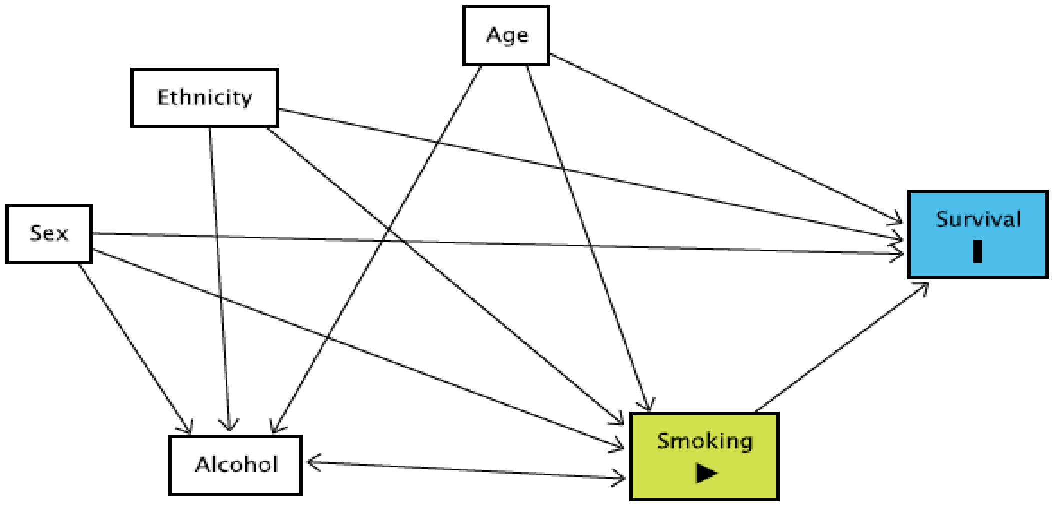

Supplementary Figure S4: Potential confounders of the association between smoking and FL survival

Evidence underpinning associations included in directed acyclic graph (Supplementary Figure S4):

Smoking status and age – Smoking varies by age (1)

Smoking status and sex – Higher smoking prevalence in men compared to women (2)

Smoking status and ethnicity – Higher smoking prevalence among white compared to black population (3)

Smoking – Poorer FL survival with smoking (13)

Age – Higher FL survival among age group <60 years (14)

Sex – Higher FL survival in males than females (14)

Ethnicity – Higher FL survival among Caucasians compared to other ethnic groups (14)

Alcohol intake and smoking – Smoking is significantly related to alcohol use (7, 8)

Alcohol intake and age – Alcohol intake varies by age (9)

Alcohol intake and sex – Alcohol intake higher in men than women (10)

Alcohol intake and ethnicity – Alcohol intake varies by ethnicity (11, 12)

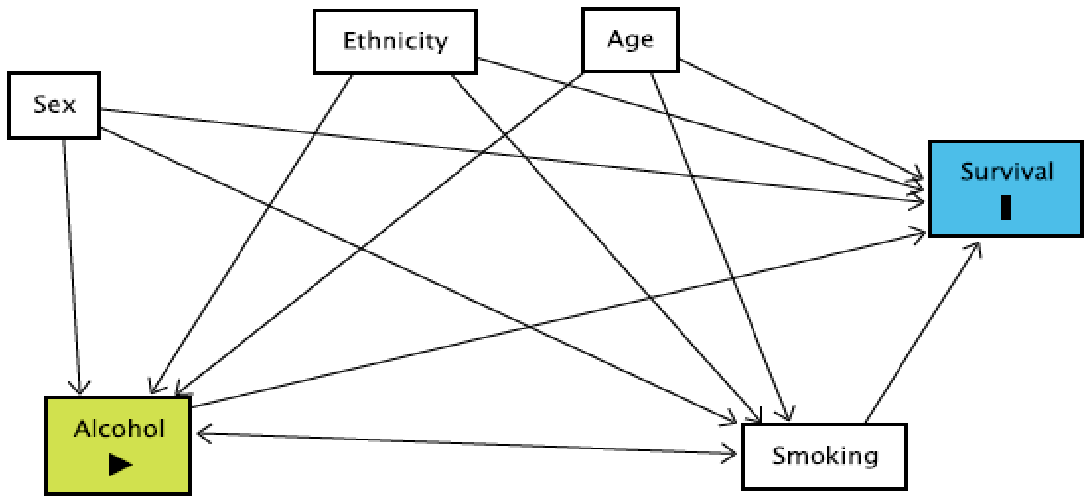

Supplementary Figure S5: Potential confounders of the association between alcohol and FL survival

Evidence underpinning associations included in directed acyclic graph (Supplementary Figure S5):

As for Supplementary Figure 4.

SUPPLEMENTARY TABLES

Supplementary Table S1: Odds ratios and 95% confidence intervals for FL risk in relation to smoking in cases and unrelated controls

Exposures	Cases	Reference category included passive smokers			Cases	Reference category excluded passive smokers		
		Unrelated controls	OR (95% CI)[a]	*P*		Unrelated controls	OR (95% CI)[a]	*P*
Smoking status								
Never	369	118	Ref.	0.01	110	35	Ref.	0.03
Ever	340	69	1.66 (1.17-2.35)		340	69	1.76 (1.06-2.92)	
Smoking status								
Never	369	118	Ref.	0.02	110	35	Ref.	0.07
Former	274	56	1.73 (1.19-2.52)		274	56	1.87 (1.10-3.16)	
Current	66	13	1.38 (0.72-2.62)		66	13	1.41 (0.68-2.94)	
Age started smoking[b]								
Never	369	118	Ref.	0.05	110	35	Ref.	0.18
Tertile 1 (>18)	97	19	1.58 (0.92-2.75)		97	19	1.68 (0.89-3.18)	
Tertile 2 (17-18)	110	25	1.59 (0.93-2.61)		110	25	1.70 (0.87-3.30)	
Tertile 3 (<17)	132	25	1.78 (1.07-2.95)		132	25	1.87 (1.01-3.47)	
			P_{trend} 0.02				P_{trend} 0.04	
Years since quitting smoking								
Never	369	118	Ref.	0.03	110	35	Ref.	0.10
Tertile 1 (≥30)	95	22	1.50 (0.88-2.57)		95	22	1.64 (0.85-3.18)	
Tertile 2 (15-29)	99	21	1.62 (0.94-2.81)		99	21	1.70 (0.86-3.36)	
Tertile 3 (<15)	80	13	2.29 (1.17-4.49)		80	13	2.53 (1.17-5.46)	
			P_{trend} 0.03				P_{trend} 0.05	
No. of cigarettes per day[b]								
Never	369	118	Ref.	0.02	110	35	Ref.	0.08
<10	90	17	1.66 (0.95-2.92)		90	17	1.83 (0.95-3.53)	

10-19	105	24	1.49 (0.89-2.50)		105	24	1.61 (0.86-3.02)	
≥20	132	25	1.90 (1.14-3.16)		132	25	2.06 (1.10-3.85)	
			P_{trend} 0.06				P_{trend} 0.06	
Duration of cigarette smoking (years)[b]								
Never	369	118	Ref.	0.01	110	35	Ref.	0.03
Tertile 1 (≤13)	114	28	1.29 (0.80-2.08)		114	28	1.33 (0.73-2.42)	
Tertile 2 (14-27)	106	16	1.52 (0.92-2.50)		106	16	1.61 (0.86-3.02)	
Tertile 3 (>27)	119	25	2.76 (1.45-5.24)		119	25	3.09 (1.47-6.50)	
			P_{trend} 0.01				P_{trend} 0.04	
Lifetime cigarette exposure (pack-years)								
Never	369	118	Ref.	0.04	110	35	Ref.	0.16
Tertile 1 (<6.8)	111	21	1.69 (0.98-2.89)		111	21	1.77 (0.95-3.33)	
Tertile 2 (6.8-19.9)	102	22	1.48 (0.87-2.52)		102	22	1.61 (0.84-3.06)	
Tertile 3 (≥20.0)	113	23	1.78 (1.05-3.02)		113	23	1.87 (0.98-3.59)	
			P_{trend} 0.01				P_{trend} 0.08	

[a] Multivariable model – adjusted for age, sex, ethnicity, state, quantity of alcohol intake 12 months prior to enrolment.

[b] Imputations (number of participants with missing values): age started smoking (1), number of cigarettes per day (11), duration of smoking (1).

Supplementary Table S2: Odds ratios and 95% confidence intervals for FL risk in relation to smoking in cases and related (sibling) controls

Exposures	Cases	Reference category included passive smokers			Cases	Reference category excluded passive smokers		
		Related controls	OR (95% CI)[a]	*P*		Related controls	OR (95% CI)[a]	*P*
Smoking status[b]								
Never	130	175	Ref.	0.43	48	68	Ref.	0.69
Ever	112	127	1.19 (0.78-1.81)		112	127	1.16 (0.57-2.35)	
Smoking status[b]								
Never	130	175	Ref.	0.43	48	68	Ref.	0.92
Former	88	106	1.11 (0.71-1.73)		88	106	1.15 (0.55-2.38)	
Current	24	21	1.68 (0.76-3.72)		24	21	1.21 (0.40-3.62)	
Age started smoking								
Never	130	175	Ref.	0.80	48	68	Ref.	0.59
Tertile 1 (>18)	33	29	1.04 (0.62-1.76)		33	29	0.97 (0.42-2.22)	
Tertile 2 (17-18)	43	54	1.06 (0.65-1.73)		43	54	1.07 (0.45-2.55)	
Tertile 3 (<17)	36	44	1.35 (0.75-2.42)		36	44	1.82 (0.67-4.93)	
			P_{trend} 0.39				P_{trend} 0.39	
Years since quitting smoking[b]								
Never	130	175	Ref.	0.61	48	68	Ref.	0.62
Tertile 1 (≥30)	32	33	1.38 (0.73-2.60)		32	33	1.81 (0.61-5.37)	
Tertile 2 (15-29)	29	34	0.98 (0.48-2.01)		29	34	1.01 (0.35-2.92)	
Tertile 3 (<15)	27	39	0.74 (0.35-1.58)		27	39	0.79 (0.27-2.34)	
			P_{trend} 0.44				P_{trend} 0.33	
No. of cigarettes per day[b]								
Never	130	175	Ref.	0.09	48	68	Ref.	0.32
<10	39	28	1.88 (0.95-3.71)		39	28	1.46 (0.58-3.71)	
10-19	26	49	0.66 (0.34-1.28)		26	49	0.65 (0.25-1.69)	

≥20	42	45	1.24 (0.72-2.16)		43	45	1.44 (0.61-3.40)	
			P_{trend} 0.76				P_{trend} 0.68	
Duration of cigarette smoking (years)[b]								
Never	130	175	Ref.	0.79	48	68	Ref.	0.74
Tertile 1 (≤13)	47	45	1.04 (0.55-1.95)		47	45	1.19 (0.53-2.63)	
Tertile 2 (14-27)	35	49	1.22 (0.72-2.08)		35	49	1.56 (0.57-4.25)	
Tertile 3 (>27)	30	33	1.35 (0.66-2.74)		30	33	0.77 (0.27-2.16)	
			P_{trend} 0.45				P_{trend} 0.38	
Lifetime cigarette exposure (pack-years)								
Never	130	175	Ref.	0.14	48	68	Ref.	0.38
Tertile 1 (<6.8)	46	40	1.53 (0.84-2.80)		46	40	1.47 (0.59-3.64)	
Tertile 2 (6.8-19.9)	28	49	0.78 (0.41-1.47)		28	49	0.73 (0.29-1.78)	
Tertile 3 (≥20.0)	33	33	1.65 (0.81-3.34)		33	33	1.61 (0.58-4.47)	
			P_{trend} 0.38				P_{trend} 0.72	

[a] Multivariable model – adjusted for age, sex, ethnicity, state, quantity of alcohol intake 12 months prior to enrolment.

[b] Imputations (number of participants with missing values): ever smoking status (1), year since quit smoking (1), number of cigarettes per day (12), duration of smoking (1).

Supplementary Table S3: Odds ratios and 95% confidence intervals for FL risk in relation to passive smoking exposure among never smokers: cases and unrelated controls

Passive smoking	Cases	Unrelated controls	OR (95% CI)[a]	*P*
Never smokers with no passive smoking exposure	109	35	Ref.	
Childhood only passive smoking[b]				
Intensity (no. of smokers)[b]				
1	109	37	0.94 (0.54-1.66)	0.25
2	46	19	0.79 (0.39-1.59)	
>2	67	13	1.81 (0.84-3.86)	
Duration (years)[b]				
1-6	72	26	0.81 (0.44-1.50)	0.52
7-10	67	17	1.18 (0.60-2.35)	
>10	73	23	1.01 (0.53-1.94)	
Adulthood only passive smoking[b]				
Intensity (no. of smokers)[b]				
1	41	17	0.94 (0.46-1.94)	0.98
2-4	55	15	1.08 (0.54-2.16)	
>4	65	18	1.16 (0.57-2.38)	
			P_{trend} 0.72	
Duration (years)[b]				
≤6	35	17	0.72 (0.34-1.53)	0.61
7-18	60	16	1.17 (0.57-2.38)	
>18	58	14	1.42 (0.67-3.02)	
			P_{trend} 0.29	
Childhood and adulthood passive smoking[b]	259	83	0.97 (0.59-1.58)	0.89
Social venue passive smoking as an adult[b]				
Duration (years)[b]				
≤2	56	17	1.29 (0.62-2.68)	0.32
>2	29	4	2.43 (0.74-7.90)	
			P_{trend} 0.14	

[a] Multivariable model – adjusted for age, sex, ethnicity, state, quantity of alcohol intake 12 months prior to enrolment.

[b] Imputations (number of participants with missing values): childhood passive smoking – intensity (6), duration (18); adulthood – intensity (3), duration (11); childhood or adulthood (2); social venues: duration (5).

Supplementary Table S4: Odds ratios and 95% confidence intervals for FL risk in relation to passive smoking exposure among never smokers: cases and related (sibling) controls

Passive smoking	Cases	Related controls	OR (95% CI)[a]	*P*
Never smokers with no passive smoking exposure	48	68	Ref.	
Childhood only passive smoking[b]				
Intensity (no. of smokers)[b]				
1	31	38	0.95 (0.21-4.19)	0.56
2	19	28	0.34 (0.14-2.60)	
>2	17	21	1.27 (0.26-6.31)	
Duration (years)[b]				
1-6	24	25	0.99 (0.23-4.33)	0.79
7-10	17	31	0.46 (0.16-3.36)	
>10	25	31	1.05 (0.18-6.19)	
Adulthood only passive smoking[b]				
Intensity (no. of smokers)[b]				
1	15	23	1.15 (0.27-4.83)	0.81
2-4	18	31	1.84 (0.45-7.57)	
>4	19	21	1.89 (0.44-7.59)	
			P_{trend} 0.37	
Duration (years)[b]				
≤6	14	19	1.66 (0.39-7.30)	0.49
7-18	24	27	2.28 (0.48-7.88)	
>18	11	28	0.84 (0.21-3.28)	
Childhood and adulthood passive smoking[b]	82	108	1.24 (0.47-3.28)	0.67
Social venue passive smoking as an adult[b]				
Duration (years)[b]				
≤2	22	20	1.22 (0.32-4.61)	0.69
>2	14	19	1.61 (0.55-4.71)	
			P_{trend} 0.38	

[a] Multivariable model – adjusted for age, sex, ethnicity, state, quantity of alcohol intake 12 months prior to enrolment.

[b] Imputations (number of participants with missing values): childhood passive smoking – intensity (5), duration (6); adulthood – intensity (5), duration (9); childhood or adulthood (4); social venues: duration (6).

Supplementary Table S5: Odds ratios and 95% confidence intervals for FL risk in relation to alcohol intake 12 months prior to enrolment by control types

Exposures	Cases	Unrelated controls	OR (95% CI)[a]	*P*	Cases	Related controls	OR (95% CI)[a]	*P*
Alcohol intake								
No	79	24	Ref.	0.89	27	31	Ref.	0.91
Yes	630	163	1.04 (0.62-1.74)		215	272	1.04 (0.50-2.17)	
Beer intake								
No	232	75	Ref.	0.47	89	125	Ref.	0.38
Yes	398	88	0.78 (0.40-1.52)		126	147	0.53 (0.13-2.20)	
Wine intake								
No	82	15	Ref.	0.92	19	28	Ref.	0.78
Yes	548	148	1.05 (0.39-2.82)		196	244	1.18 (0.34-3.92)	
Spirit intake								
No	274	70	Ref.	0.78	100	121	Ref.	0.37
Yes	356	93	0.93 (0.54-1.60)		115	151	0.57 (0.17-1.91)	

[a] Multivariable model – adjusted for age, sex, ethnicity, state, smoking (never, current, former); estimates of beer, wine, and spirits intake were mutually adjusted for each other.

Supplementary Table S6: Hazard ratios and 95% confidence intervals for all-cause mortality and FL-specific mortality in relation to smoking with further adjustment for stage of disease and first-line treatment

Exposures	No. of deaths/person- months	Reference category included passive smoking		Reference category excluded passive smokers	
		HR (95% CI)[a]	P	HR (95% CI)[a]	P
All-cause mortality					
Smoking status					
Never	18/31022	Ref.	0.13	Ref.	0.34
Ever	31/27802	1.59 (0.88-2.90)		1.61 (0.61-4.23)	
Smoking status					
Never	18/31022	Ref.	0.01	Ref.	0.01
Former	20/5230	1.22 (0.64-2.34)		1.20 (0.44-3.26)	
Current	11/22572	3.93 (1.79-8.62)		3.77 (1.28-11.15)	
Age started smoking (years)					
Never	18/31022	Ref.	0.49	Ref.	0.66
≥18	16/14022	1.54 (0.75-3.15)		1.57 (0.55-4.43)	
<18	15/13698	1.71 (0.86-3.39)		1.69 (0.61-4.69)	
Years since quitting smoking					
Never	18/31022	Ref.	0.15	Ref.	0.15
≥20	10/13883	0.91 (0.41-2.03)		0.92 (0.41-2.04)	
<20	10/8689	1.89 (0.86-4.12)		1.90 (0.87-4.17)	
No. of cigarettes per day[b]					
Never	18/31022	Ref.		Ref.	
Former smokers					
<20	9/13103	0.95 (0.42-2.14)	0.23	0.96 (0.31-2.94)	0.24
≥20	10/8860	1.50 (0.68-3.31)		1.48 (0.49-4.45)	
Current smokers					
<20	7/3073	5.54 (2.25-13.65)	<0.01	5.00 (1.56-16.09)	0.01
≥20	4/1766	2.94 (0.86-10.09)		2.98 (0.70-12.71)	
Duration of cigarette smoking (years)					
Never	18/31022	Ref.	<0.01	Ref.	<0.01
Tertile 1 (≤13)	4/9507	0.52 (0.15-1.77)		0.54 (0.13-2.27)	
Tertile 2 (14-27)	5/8783	0.88 (0.33-2.39)		0.92 (0.26-3.22)	
Tertile 3 (>27)	22/9430	3.23 (1.69-6.19)		3.39 (1.24-9.29)	
		P_{trend} <0.01		P_{trend} <0.01	
Lifetime cigarette exposure (pack-years)[b]					
Never	18/31022	Ref.	0.02	Ref.	0.38
Tertile 1 (<6.8)	5/9005	0.81 (0.28-2.34)		0.79 (0.21-2.94)	
Tertile 2 (6.8-19.9)	8/8628	1.44 (0.62-3.36)		1.51 (0.48-4.70)	
Tertile 3 (≥20.0)	17/9088	2.52 (1.27-4.99)		2.48 (0.90-6.86)	
		P_{trend} 0.01		P_{trend} 0.09	
FL-specific mortality					
Smoking status					

Never	9/31022	Ref.	0.46	Ref.	0.50
Ever	14/27802	1.39 (0.58-3.32)		1.68 (0.36-7.69)	
Smoking status					
Never	9/31022	Ref.	0.26	Ref.	0.26
Former	9/22572	0.85 (0.29-2.55)		1.11 (0.43-2.84)	
Current	5/5230	2.65 (0.68-10.35)		2.65 (0.68-10.35)	
Lifetime cigarette exposure (pack-years)[b]					
Never	9/31022	Ref.	0.53	Ref.	0.65
<20	7/17633	1.06 (0.37-3.04)		1.09 (0.38-3.11)	
≥20	6/9088	1.69 (0.58-4.89)		1.85 (0.67-5.13)	

[a] Multivariable model – adjusted for age, sex, ethnicity, state, stage of disease and first-line treatment.

[b] Imputations (number of participants with missing values): no. of cigarettes per day (13), duration (1), pack-years (14), stage (179), treatment (174).

Supplementary Table S7: Odds ratios and 95% confidence intervals for FL risk in relation to personal smoking after excluding cases with grade 3B histology and composite follicular lymphoma/diffuse large B-cell lymphoma

Exposures	Cases	Reference category included passive smokers				Reference category excluded passive smokers			
		Related controls	Unrelated controls	OR (95% CI)[a]	*P*	Related controls	Unrelated controls	OR (95% CI)[a]	*P*
Smoking status[b]									
Never	330	175	118	Ref.	0.01	68	35	Ref.	0.02
Ever	311	127	69	1.39 (1.09-1.78)		127	69	1.52 (1.08-2.12)	
Smoking status[b]									
Never	330	175	118	Ref.	0.03			Ref.	0.05
Former	250	106	56	1.38 (1.06-1.81)		106	56	1.51 (1.05-2.16)	
Current	6	21	13	1.44 (0.92-2.24)		21	13	1.55 (0.95-2.54)	
Age started smoking[b]									
Never	330	175	118	Ref.	0.06	68	35	Ref.	0.08
Tertile 1 (>18)	86	29	19	1.47 (0.98-2.20)		29	19	1.59 (1.00-2.54)	
Tertile 2 (17-18)	103	54	25	1.24 (0.87-1.76)		54	25	1.37 (0.90-2.09)	
Tertile 3 (<17)	121	44	25	1.48 (1.06-2.09)		44	25	1.60 (1.06-2.42)	
				P_{trend} 0.17				P_{trend} 0.07	
Years since quitting smoking[b]									
Never	330	175	118	Ref.	0.10	68	35	Ref.	0.18
Tertile 1 (≥30)	89	33	22	1.56 (0.96-2.13)		33	22	1.64 (0.98-2.67)	
Tertile 2 (15-29)	91	34	21	1.41 (0.96-2.06)		34	21	1.52 (0.95-2.41)	
Tertile 3 (<15)	70	39	13	1.19 (0.80-1.76)		39	13	1.33 (0.84-2.10)	
				P_{trend} 0.05				P_{trend} 0.05	
No. of cigarettes per day[b]									
Never	330	175	118	Ref.	0.05	68	35	Ref.	0.02
<10	85	28	17	1.56 (1.04-2.35)		28	17	1.87 (1.17-3.00)	
10-19	94	49	24	1.16 (0.81-1.66)		49	24	1.29 (0.84-1.98)	

≥20	119	45	25	1.42 (1.01-1.99)		45	25	1.57 (1.04-2.36)	
				P_{trend} 0.06				P_{trend} 0.09	
Duration of cigarette smoking (years)[b]									
Never	330	175	118	Ref.	0.05	68	35	Ref.	0.08
Tertile 1 (≤13)	108	45	28	1.30 (0.91-1.87)		45	28	1.43 (0.92-2.20)	
Tertile 2 (14-27)	95	49	16	1.41 (0.97-2.04)		49	16	1.57 (1.01-2.43)	
Tertile 3 (>27)	107	33	25	1.49 (1.04-2.14)		33	25	1.58 (1.01-2.43)	
				P_{trend} 0.01				P_{trend} 0.03	
Lifetime cigarette exposure (pack-years)[b]									
Never	330	175	118	Ref.	0.05	68	35	Ref.	0.14
Tertile 1 (<6.8)	104	40	21	1.49 (0.99-2.16)		40	21	1.69 (1.09-2.63)	
Tertile 2 (6.8-19.9)	93	49	22	1.16 (0.80-1.67)		49	22	1.33 (0.85-2.06)	
Tertile 3 (≥20.0)	100	33	23	1.50 (1.04-2.15)		33	23	1.57 (1.02-2.43)	
				P_{trend} 0.05				P_{trend} 0.06	

[a] Multivariable model – adjusted for age, sex, ethnicity, state, quantity of alcohol intake 12 months prior to enrolment. ORs are based on related and unrelated controls combined.

[b] Imputations (number of participants with missing values): ever smoking status (1), age started smoking (1), year since quit smoking (1), number of cigarettes per day (22), duration of smoking (2), pack-years (23).

Supplementary Table S8: Odds ratios and 95% confidence intervals for FL risk in relation to passive smoking exposure among never smokers after excluding cases with grade 3B histology and composite follicular lymphoma/diffuse large B-cell lymphoma

Passive smoking	Cases	Related controls	Unrelated controls	OR (95% CI)[a]	*P*
Never smokers with no passive smoking exposure	109	68	35	Ref.	
Childhood only passive smoking[b]					
Intensity (no. of smokers)[b]					
1	96	38	37	1.18 (0.78-1.77)	0.04
2	39	28	19	0.78 (0.48-1.29)	
>2	63	21	13	1.89 (1.14-3.13)	
				P_{trend} 0.09	
Duration (years)[b]					
1-6	64	25	26	1.22 (0.76-1.95)	0.71
7-10	56	31	17	1.12 (0.69-1.80)	
>10	68	31	23	1.20 (0.77-1.89)	
Adulthood only passive smoking[b]					
Intensity (no. of smokers)[b]					
1	37	23	17	0.92 (0.51-1.66)	0.31
2-4	43	31	15	0.88 (0.51-1.51)	
>4	59	21	18	1.46 (0.88-2.44)	
				P_{trend} 0.28	
Duration (years)[b]					
≤6	28	19	17	0.79 (0.44-1.43)	0.57
7-18	51	27	16	1.17 (0.70-1.95)	
>18	52	28	14	1.30 (0.75-2.24)	
				P_{trend} 0.27	
Childhood and adulthood passive smoking[b]	230	108	83	1.17 (0.83-1.65)	0.37
Social venue passive smoking as an adult[b]					
Duration (years)[b]					
≤2	51	20	17	1.40 (0.81-2.42)	0.36
>2	24	19	4	1.00 (0.47-2.15)	

[a] Multivariable model – adjusted for age, sex, ethnicity, state, quantity of alcohol intake 12 months prior to enrolment. ORs are based on related and unrelated controls combined.

[b] Imputations (number of participants with missing values): childhood passive smoking – intensity (11), duration (24); adulthood – intensity (8), duration (20); childhood or adulthood (6); social venues – duration (11).

Supplementary Table S9: Odds ratios and 95% confidence intervals for FL risk in relation to alcohol intake 12 months prior to enrolment after excluding cases with grade 3B histology and composite follicular lymphoma/diffuse large B-cell lymphoma

Exposures	Cases	Related controls	Unrelated controls	OR (95% CI)[a]	*P*
Alcohol intake					
No	74	31	24	Ref.	0.82
Yes	567	272	163	0.96 (0.65-1.40)	
Frequency of any alcohol intake (per week)					
None	74	31	24	Ref.	0.50
< once	166	82	44	1.04 (0.68-1.61)	
once	58	21	17	1.14 (0.64-2.01)	
> once	343	169	102	0.89 (0.60-1.31)	
Quantity of any alcohol intake (grams of ethanol/day)[b]					
None	74	31	24	Ref.	0.15
>5.20	193	94	58	1.08 (0.70-1.66)	
5.20-19.70	196	80	58	1.03 (0.67-1.58)	
>19.70	177	98	46	0.76 (0.50-1.17)	
				P_{trend} 0.10	
Beer intake					
No	208	125	75	Ref.	0.84
Yes	359	147	88	1.04 (0.74-1.45)	
Frequency of beer intake (per week)					
None	208	125	75	Ref.	0.97
< once	160	72	39	1.04 (0.72-1.51)	
once	50	18	16	0.93 (0.53-1.64)	
> once	149	57	33	1.06 (0.69-1.62)	
Quantity of beer intake (grams of ethanol/day)[b]					
None	208	125	75	Ref.	0.90
<1.46	133	61	33	1.07 (0.73-1.57)	
1.46-7.76	103	33	30	0.93 (0.59-1.46)	
>7.76	121	53	25	0.92 (0.58-1.47)	
				P_{trend} 0.66	
Quantity of beer that was light beer[b]					
None	208	125	75	Ref.	0.37
Almost none	173	73	45	0.90 (0.61-1.33)	
Less than half	35	19	8	079 (0.42-1.48)	
About half	42	19	8	1.03 (0.57-1.88)	
More than half	13	9	3	0.55 (0.22-1.37)	
All or almost all	90	27	23	1.28 (0.82-2.01)	
Wine intake					
No	78	28	15	Ref.	0.39
Yes	489	244	148	0.83 (0.55-1.26)	
Frequency of wine intake (per week)					
None	78	28	15	Ref.	0.33

< once	173	79	44	0.96 (0.59-1.54)	
once	63	22	14	1.21 (0.68-2.18)	
> once	253	143	90	0.71 (0.46-1.08)	
Quantity of wine intake (grams of ethanol/day)[b]					
None	78	28	15	Ref.	0.17
<2.98	205	84	55	1.05 (0.66-1.67)	
2.98-14.49	163	77	53	0.84 (0.53-1.31)	
>14.49	121	83	39	0.63 (0.39-1.02)	
				P_{trend} 0.10	
Quantity of wine that was red wine[b]					
None	78	28	15	Ref.	0.68
Almost none	129	66	55	0.80 (0.50-1.29)	
Less than half	69	34	19	0.84 (0.49-1.44)	
About half	93	46	26	0.84 (0.50-1.39)	
More than half	46	31	12	0.64 (0.36-1.14)	
All or almost all	152	67	35	0.04 (0.59-1.50)	
Spirit intake					
No	250	121	70	Ref.	0.94
Yes	317	151	93	1.01 (0.78-1.32)	
Frequency of spirit intake (per week)					
None	250	121	70	Ref.	0.99
< once	231	119	67	1.00 (0.75-1.33)	
once	36	12	8	1.28 (0.71-2.32)	
> once	50	20	18	0.92 (0.56-1.50)	
Quantity of spirit intake (grams of ethanol/day)					
None	250	121	70	Ref.	0.98
<0.24	117	61	35	0.99 (0.70-1.40)	
0.24-1.23	99	48	26	1.06 (0.71-1.58)	
>1.23	101	42	32	0.99 (0.68-1.45)	

[a] Multivariable model – adjusted for age, sex, ethnicity, state, smoking (never, current, former); estimates of beer, wine, and spirits intake were mutually adjusted for each other. ORs are based on related and unrelated controls combined.

[b] Imputations (number of participants with missing values): quantity of beer (2), light beer (7), quantity of wine (1), and red wine intake (1).

Supplementary Table S10: Hazard ratios and 95% confidence intervals for all-cause mortality and FL-specific mortality in relation to smoking after excluding cases with grade 3B histology and composite follicular lymphoma/diffuse large B-cell lymphoma

Exposures	No. of deaths/person- months	Reference category included passive smoking		Reference category excluded passive smokers	
		HR (95% CI)[a]	*P*	HR (95% CI)[a]	*P*
All-cause mortality					
Smoking status					
Never	16/27919	Ref.	0.10	Ref.	0.43
Ever	29/25492	1.68 (0.90-3.14)		1.47 (0.56-3.85)	
Smoking status					
Never	16/27919	Ref.	<0.01	Ref.	0.01
Former	19/20643	1.32 (0.68-2.57)		1.13 (0.42-3.06)	
Current	10/4848	3.76 (1.68-8.41)		3.33 (1.11-10.00)	
Age started smoking (years)					
Never	16/27919	Ref.	0.18	Ref.	0.44
≥18	16/12731	1.47 (0.69-3.10)		1.28 (0.44-3.66)	
<18	13/12680	1.91 (0.96-3.80)		1.67 (0.60-4.61)	
Years since quitting smoking					
Never	16/27919	Ref.	0.11	Ref.	0.11
≥20	10/13023	0.97 (0.44-2.15)		0.97 (0.44-2.15)	
<20	9/7620	2.21 (0.89-4.92)		2.21 (0.88-4.92)	
No. of cigarettes per day[b]					
Never	16/27919	Ref.		Ref.	
Former smokers					
<20	9/12073	1.16 (0.51-2.63)	0.48	1.02 (0.35-2.99)	0.67
≥20	9/7961	1.67 (0.73-3.80)		1.48 (0.51-4.33)	
Current smokers					
<20	6/2835	4.48 (1.80-11.12)	<0.01	4.58 (1.33-15.80)	0.04
≥20	4/1623	3.35 (0.97-11.55)		3.72 (0.79-17.60)	
Duration of cigarette smoking (years)					
Never	16/27919	Ref.	<0.01	Ref.	<0.01
Tertile 1 (≤13)	4/9038	0.58 (0.17-2.00)		0.52 (0.12-2.18)	
Tertile 2 (14-27)	4/7884	0.85 (0.28-2.56)		0.77 (0.21-2.89)	
Tertile 3 (>27)	21/8488	3.41 (1.77-6.56)		3.07 (1.12-8.45)	
		P_{trend} <0.01		P_{trend} <0.01	
Lifetime cigarette exposure (pack-years)[b]					
Never	16/27919	Ref.	0.04	Ref.	0.24
Tertile 1 (<6.8)	5/8462	0.87 (0.29-2.56)		0.70 (0.19-2.62)	
Tertile 2 (6.8-19.9)	8/7920	1.70 (0.72-4.05)		1.45 (0.47-4.46)	
Tertile 3 (≥20.0)	15/8029	2.49 (1.22-5.07)		2.10 (0.75-5.85)	
		P_{trend} 0.01		P_{trend} 0.05	
FL-specific mortality					
Smoking status					

Never	7/27919	Ref.	0.21	Ref.	0.77
Ever	14/25492	1.82 (0.72-4.61)		1.81 (0.40-8.12)	
Smoking status					
Never	7/27919	Ref.	0.18	Ref.	0.18
Former	9/16382	1.48 (0.55-4.04)		1.48 (0.55-4.04)	
Current	5/8029	3.52 (0.94-12.09)		3.52 (0.94-12.09)	
Lifetime cigarette exposure (pack-years)[b]					
Never	7/27919	Ref.	0.43	Ref.	0.26
<20	7/17633	1.42 (0.41-4.93)		1.43 (0.48-4.26)	
≥20	6/9088	2.26 (0.70-7.30)		2.44 (0.83-7.15)	

[a] Basic model – adjusted for age, sex, ethnicity, state.

[b] Imputations (number of participants with missing values): no. of cigarettes per day (13), duration (1), pack-years (14).

Supplementary Table S11: Hazard ratios and 95% confidence intervals for all-cause mortality after FL diagnosis in relation to passive smoking exposure among never smokers after excluding cases with grade 3B histology and composite follicular lymphoma/diffuse large B-cell lymphoma

Passive smoking	Person-months	No. of deaths	All-cause mortality	
			HR (95% CI)[a]	*P*
Never smokers with no passive smoking exposure	8256	5	Ref.	
Childhood only passive smoking				
Intensity (no. of smokers)				
<2	8448	5	0.85 (0.24-2.99)	0.96
≥2	8599	5	0.97 (0.28-3.39)	
Duration (years)				
<7	5422	3	0.77 (0.18-3.31)	0.90
≥7	10644	7	1.06 (0.33-3.37)	
Adulthood only passive smoking				
Intensity (no. of smokers)				
≤4	6764	5	1.14 (0.32-4.09)	0.78
>4	4946	5	1.56 (0.44-5.52)	
Duration (years)[b]				
≤18	6670	5	1.29 (0.39-4.34)	0.80
>18	4460	4	1.46 (0.37-5.72)	
Childhood and adulthood passive smoking	21365	13	0.95 (0.33-2.69)	0.92
Social venues passive smoking	10786	6	0.85 (0.30-2.40)	0.76

[a] Basic model – adjusted for age, sex, ethnicity, state.

[b] Imputations (number of participants with missing values): adulthood passive smoking – duration (1).

References

1. Substance Abuse and Mental Health Services Administration (SAMHSA). 2019 National Survey on Drug Use and Health (NSDUH). Table 2.1B—Tobacco product and alcohol use in lifetime, past year, and past month among persons aged 12 or older, by age group: percentages, 2018 and 2019. https://www.samhsa.gov/data/sites/default/files/cbhsq-reports/NSDUHDetailedTabs2018R2/NSDUHDetTabsSect2pe2018.htm#tab2-1b. Accessed 05 April 2021.
2. Peters SAE, Huxley RR, Woodward M. Do smoking habits differ between women and men in contemporary Western populations? Evidence from half a million people in the UK Biobank study. BMJ open. 2014;4(12):e005663-e63.
3. El-Toukhy S, Sabado M, Choi K. Trends in susceptibility to smoking by race and ethnicity. Pediatrics. 2016;138(5):e20161254.
4. Jayasekara H, Karahalios A, Juneja S, Thursfield V, Farrugia H, English DR, *et al.* Incidence and survival of lymphohematopoietic neoplasms according to the World Health Organization classification: a population-based study from the Victorian Cancer Registry in Australia. Leuk Lymphoma. 2010;51(3):456-68.
5. Morton LM, Wang SS, Devesa SS, Hartge P, Weisenburger DD, Linet MS. Lymphoma incidence patterns by WHO subtype in the United States, 1992-2001. Blood. 2006;107(1):265-76.
6. Odutola MK, Nnakelu E, Giles GG, van Leeuwen MT, Vajdic CM. Lifestyle and risk of follicular lymphoma: a systematic review and meta-analysis of observational studies. Cancer Causes Control. 2020;31(11):979-1000.
7. Friedman GD, Tekawa I, Klatsky AL, Sidney S, Armstrong MA. Alcohol drinking and cigarette smoking: an exploration of the association in middle-aged men and women. Drug Alcohol Depend. 1991;27(3):283-90.
8. De Leon J, Rendon DM, Baca-Garcia E, Aizpuru F, Gonzalez-Pinto A, Anitua C, *et al.* Association between smoking and alcohol use in the general population: stable and unstable odds ratios across two years in two different countries. Alcohol Alcohol. 2007;42(3):252-7.
9. Substance Abuse and Mental Health Services Administration (SAMHSA). 2019 National Survey on Drug Use and Health (NSDUH). Table 5.4A—Alcohol use disorder in past year

among persons aged 12 or older, by age group and demographic characteristics: numbers in thousands, 2018 and 2019. https://www.samhsa.gov/data/sites/default/files/cbhsq-reports/NSDUHDetailedTabs2018R2/NSDUHDetTabsSect5pe2018.htm#tab5-4a. Accessed 05 April 2021.

10. Wilsnack RW, Wilsnack SC, Kristjanson AF, Vogeltanz-Holm ND, Gmel G. Gender and alcohol consumption: patterns from the multinational GENACIS project. Addiction. 2009;104(9):1487-500.
11. Rao R, Schofield P, Ashworth M. Alcohol use, socioeconomic deprivation and ethnicity in older people. BMJ Open. 2015;5(8):e007525.
12. Jorgenson E, Thai KK, Hoffmann TJ, Sakoda LC, Kvale MN, Banda Y, *et al.* Genetic contributors to variation in alcohol consumption vary by race/ethnicity in a large multi-ethnic genome-wide association study. Mol Psychiatry. 2017;22(9):1359-67.
13. Geyer SM, Morton LM, Habermann TM, Allmer C, Davis S, Cozen W, *et al.* Smoking, alcohol use, obesity, and overall survival from non-Hodgkin lymphoma: a population-based study. Cancer. 2010;116(12):2993-3000.
14. Bista A, Sharma S, Shah BK. Disparities in receipt of radiotherapy and survival by age, sex, and ethnicity among patient with stage I follicular lymphoma. Front Oncol. 2016;6:101-01.
